# Molecular Characterization of Neurogranin (*NRGN*) Gene from Red‑Bellied Pacu (*Piaractus brachypomus*)

**DOI:** 10.1007/s12035-023-03700-5

**Published:** 2023-11-03

**Authors:** Valentina Rueda-García, Iang Schroniltgen Rondón-Barragán

**Affiliations:** Research Group in Immunobiology and Pathogenesis, Laboratory of Immunology and Molecular Biology, Faculty of Veterinary Medicine and Zootechnics, Universidad del Tolima, Building 33 L105, 730002 Ibagué, Tolima Colombia

**Keywords:** Traumatic brain injury, Organophosphate, Gene expression, Biomarker, Neurogranin, Non-conventional biomedical models

## Abstract

Neurogranin (NRGN) is a small brain protein expressed in various telencephalic areas and plays an essential role in synaptic plasticity by regulating the availability of calmodulin (CaM). The study aims to characterize the neurogranin gene in Colombian native fish, red-bellied pacu, *Piaractus brachypomus*, its basal tissue expression and differential expression in brain injury and sublethal toxicity by organophosphates. *NRGN* gene contains an open reading frame of 183 nucleotides encoding for 60 amino acids. Bioinformatics analysis showed an IQ motif necessary in the interaction with CaM. *NRGN* mRNA was detected in tissues with higher expression in brain, gills, and head kidney. In brain regions, *NRGN* showed high expression in the telencephalon (TE) and olfactory bulb (OB). In the sublethal toxicity experiment, *NRGN* mRNA was upregulated in individuals under organophosphate exposure in the OB and optic chiasm (OC). In brain injury experiment, *NRGN* showed upregulation at 14 days in OC and at 24 h and 7 days in TE. These findings demonstrate the differential expression of *NRGN* under different experimental conditions which make it a candidate for a biomarker in the brain of *P. brachypomus*.

## Introduction

In recent years, molecular biomarkers of brain injury have been studied to detect early neural and axonal damage in several animal models [1]. Some of these biomarkers include calcium-binding protein S100 beta (S100B), glial fibrillary acidic protein (GFAP), and the small neuronal protein neurogranin (NRGN) [2]. Neurogranin is a neuronal protein composed of 78 amino acids (aa) with a molecular weight of 7.6 kDa which is expressed in cerebral cortex, amygdala, caudate nucleus, hippocampus, and putamen in the central nervous system [3].

NRGN is concentrated in dendritic spines which participate in synaptic signaling by regulating the availability of calmodulin (CaM) [4]. It also acts as a substrate for the C ɣ isoform of protein kinase C (PKC) by binding to CaM and plays an important role in long-term potentiation by modulating CaM signal transduction in response to intracellular calcium to enhance synaptic plasticity. Furthermore, NRGN has been shown to be a useful biomarker in neurodegenerative diseases such as Alzheimer’s disease, Parkinson’s disease, and severe traumatic brain injury (TBI) [5–7].

Studies on the function of NRGN have been carried out in various animal models. Svirsky et al. studied the effect of TBI in adult male rats undergoing controlled cortical impaction (CCI) on NRGN expression and postsynaptic density 95 in the cortex and hippocampus at 24 h, 1, 2, and 4 weeks after injury [8]. They found that the ipsilateral and contralateral hippocampus had a significant reduction in NRGN levels 1 day after CCI injury. Furthermore, NRGN levels in the ipsilateral hippocampus significantly decreased at 2 weeks after CCI injury, while they recovered to basal levels by 4 weeks. These results indicated that CCI reduces NRGN expression in a temporal- and regional-specific manner [8]. In addition, in humans with TBI, serum NRGN levels increase compared with healthy patients [7].

In fish, neurogranins have been reported with pre- and post-synaptic functions in zebrafish (*Danio rerio*) [9]. Red-bellied pacu (*Piaractus brachypomus*) is a native fish of Colombia, which has been used as a biological model in pharmacological, toxicological, and immunological studies [10–13]. Thus, the aim of this study was to characterize the *NRGN* gene in the native Colombian fish red-bellied pacu (*Piaractus brachypomus*) in models of organophosphate sublethal toxicity and brain injury.

## Materials and Methods

### Experimental Animals

For the experiments, healthy red-bellied pacu (*Piaractus brachypomus*) fingerlings (*n* = 24) were used, weighing 2.5 ± 0.3 g, regardless of sex. The fish were kept in a 90-L glass tank at a temperature of 25 °C with thermostat regulation, aeration without a filter, and feeding twice a day with a commercial concentrate equivalent to 2% of their biomass (Mojarra 30%, Solla®). The animals were acclimatized in a period of 15 days and treated with NaCl (1%) to control ectoparasites [14].

### Ethics Statement

The current study was performed according to the regulations of the Local Bioethics Committee of the Research and Scientific Development Office of the University of Tolima, based on Law 84/1989 and the Resolution 8430/1993.

### NRGN cDNA Sequencing

Sequences of *NRGN* gene were obtained from cDNA nano sequencing using MinION sequencer (Oxford Nanopore Technologies, UK) of brain of red-bellied pacu (*Piaractus brachypomus*), mapped on *Colossoma macropomum* (XP_036435715.1) as reference template, using Minimap2 algorithm on Geneious Prime software v2023.0.4 (Geneious, 2023). Primers were designed for amplifying the complete open reading frame (ORF) of *NRGN* from an assembled sequence using Geneious Prime software v2023.0.4 (Table 1). RT-PCR for *NRGN* gene from *Piaractus brachypomus* was performed from brain, liver, spleen, gill, head kidney, and blood.

**Table 1:** Sequences of primers used for PCR and qPCR assays

Gene	Primer sequence (5′-3′)	Amplicon size (bp)	Accession number	Reference
*NGRN*	F-CCACGTCGCTATGGACTGTC R-GGACGTCCTCTGAAGCTACTT	206	OQ344271	This study
*EF1a*	F-ACTGAGGTCAAGTCTGTGGA R-CCACGACGGATGTCTTTAA	110	MK085759.1	[10]

RT-PCR was performed using 1 μL of cDNA, 1 µL of dNTPs (1.5 mM) (Invitrogen, Carlsbad, CA, USA), 15.875 µL of distilled-deionized water, 1 μL of each primer (10 μM), 5 µL of 5X Colorless GoTaq® Reaction Buffer (Promega, Madison, WI, USA), and 0.125 µL of GoTaq® G2 DNA polymerase (Promega, Madison, WI, USA), in a total volume of 25 μL. The amplification was carried out in a ProFlex™ PCR System (Applied Biosystems, Carlsbad, CA, USA) with an initial denaturation step at 95 °C for 3 min, followed by 35 cycles of denaturation at 95 °C for 30 s, annealing at the specific annealing temperature for each set of primers for 30 s, extension at 72 °C for 60 s and the last step of final extension at 72 °C for 5 min. PCR products were revealed on 2% agarose gel by horizontal electrophoresis. Amplicons were sequenced by Sanger method (Macrogen Inc, Korea) and the sequences were submitted to the GenBank with accession number OQ344271. *NRGN* gene sequences were checked and identified using the web-based tool BLAST (https://blast.ncbi.nlm.nih.gov/Blast.cgi), where the percentage of identity, query cover, among others, were analyzed in different species.

### Sequence Analysis of NRGN and Model Structure Binding

From the nucleotide sequences, the amino acid (aa) sequences were deduced using the Geneious Prime v2023.0.4 software [15]. Domains were predicted using InterproScan [16] and conserved domains tool [17]. Additionally, the characteristics of the protein were analyzed using the Expasy ProtParam tool. The predicted post-translational modifications of the protein were established by the Bioinformatics Services of the Technical University of Denmark [18].

The NRGN structural model was constructed using the *Sinocyclocheilus grahami* (A0A672SZ44) model as a template from Uniprot. Amino acid mutations were performed using PyMol [19]. The CaM-NRGN complex was constructed using the complex made by Kumar et al. from *Mus musculus* (4E50) sequences, where the alignment was performed with the modified NRGN [20].

### Sequence Alignment and Phylogenetic Analysis

The genetic relationships of NRGN between different taxonomic orders of animals were performed; the amino acid (aa) sequences of the NRGN were obtained from NCBI database, as follows: *Gekko japonicus* (XP_015284890), *Sphaerodactylus townsendi* (XP_048368761) from reptiles, *Meleagris gallopavo* (XP_010721861.1), *Coturnix japonica* (XP_015739715.1) from birds, *Macaca mulatta* (NP_001254528), *Homo sapiens* (NP_001119653), *Rattus norvegicus* (NP_077054), *Canis lupus familiaris* (XP_038520483) from mammals. For teleost fish were as follows: *Alosa sapidissima* (XP_041947377), *Clupea harengus* (XP_012677423), *Alosa alosa* (XP_048093441.1) of Clupeiformes, *Astyanax mexicanus* (XP_015460274), *Pygocentrus nattereri* (XP_017552910), *Colossoma macropomum* (XP_036435715.1) of Characiformes, *Pimephales promelas* (XP_039518476), *Sinocyclocheilus grahami* (XP_016148253), *Megalobrama amblycephala* (XP_048045485.1), *Sinocyclocheilus anshuiensis* (XP_016310301.1), *Sinocyclocheilus rhinocerous* (XP_016373820.1) of Cypriniformes, *Tachysurus fulvidraco* (XP_027034393), *Ictalurus punctatus* (XP_017314959), *Silurus meridionalis* (XP_046694886), *Pangasianodon hypophthalmus* (XP_026778192) of Siluriformes, *Coregonus clupeaformis* (XP_041700919.1), *Salmo salar* (XP_013992599.1), *Oncorhynchus tshawytscha* (XP_024287894.1), *Salmo trutta* (XP_029546514.1), *Oncorhynchus mykiss* (XP_021479595.1) of Salmoniformes.

Multiple alignment of the amino acid sequences was carried out by using MUSCLE algorithm, then the phylogenetic tree was constructed by the neighbor-joining method with bootstrap values calculated from 1000 replicates using the software MEGA X [21].

### Basal Expression of *NRGN* in Tissue

Tissues were sampled from healthy *P. brachypomus* fingerlings kept under the same experimental conditions explained above. Basal expression of *NRGN* was assessed in brain, gills, liver, blood, head kidney, and spleen by RT-PCR. RT-PCR assay was performed under the same conditions as mentioned previously.

### Sublethal Exposure to Organophosphate and Brain Injury Assay

The organophosphate used was chlorpyrifos. The sublethal concentration was one tenth of the lethal concentration 50 (LC50) used in previous studies (0.011 μg/L) [22]. *P. brachypomus* fingerlings were divided into two experimental groups: fishes exposed to CPF (*n* = 3) and a control group without CPF exposure (*n* = 5). The animals were placed in a glass tank and exposed by immersion for 72 h.

For the brain injury experiment, the animals were distributed into 4 groups, as follows: 0 h (*n* = 3, control), 24 h post-lesion (*n* = 3), 7 days post-lesion (*n* = 3), and 14 days post-lesion (*n* = 3). Brain injury was performed with a 000-gauge sterile entomological needle, a deep puncture (5 mm) in the brain [23]. Immediately after injury, the animals were returned to the tank for recovery.

### Tissue Sampling, RNA Extraction, and qPCR Assay

Individuals from each experiment were immersed in a glass tank with dissolved anesthetic (Eugenol, 50 mg/L) [10] and then sacrificed by cervical dislocation [24]. Samples were snap frozen and stored in liquid nitrogen until their use.

Total RNA was extracted from all tissues using TRIzol reagent (Invitrogen, USA) following the manufacturer’s instructions. The quality of the isolated RNA was examined using the NanoDrop One (Microvolume UV–Vis Spectrophotometer, ThermoFisher Scientific, USA) and cDNA was synthesized through High Capacity cDNA Reverse Transcription kit (ThermoFisher Scientific, USA). Relative expression of *NRGN* was carried out using the same sets of primers (Table 1). The basal expression of *NRGN* was evaluated by qPCR in different regions of the brain, olfactory bulb, optic chiasm, hypothalamus, telencephalon, optic bulb, cerebellum, and medulla oblongata. In the experiments of exposure to CPF and brain injury, the expression levels of *NRGN* mRNA were assessed in the olfactory bulb, the telencephalon, and the optic chiasm. qPCR assays were performed by duplicate in the QuantStudio 3 real-time thermocycler (Applied Biosystems, USA) using 1 μL of cDNA, 8 µL of distilled-deionized water, 0.5 μL of each primer (10 μM), and 10 μL Luna® Universal qPCR Master Mix (New England Biolabs, USA), in a total volume of 20 μL. Relative gene expression was analyzed using the 2^−ΔΔCt^ method [25] and elongation factor 1-α (*EF1α*) was set as a reference gene for normalization. Data were expressed as fold change.

### Statistical Analysis

The normality of all data was evaluated using the Shapiro–Wilk test. CPF assay data was compared using the Mann–Whitney *U* test. In the brain regions relative expression and traumatic brain injury assays, for parametric data, one-way ANOVA was carried out followed by Tukey test as post hoc. In case of non-parametric data, Kruskal–Wallis test was performed followed by Dunn’s test. For all experiments, *p* < 0.05 was considered as statistically significant. All analyses were performed using GraphPad Prism v9 software for MacOS (La Jolla, CA).

## Results

### Sequence Analysis of *NRGN*

**Fig. 1:**
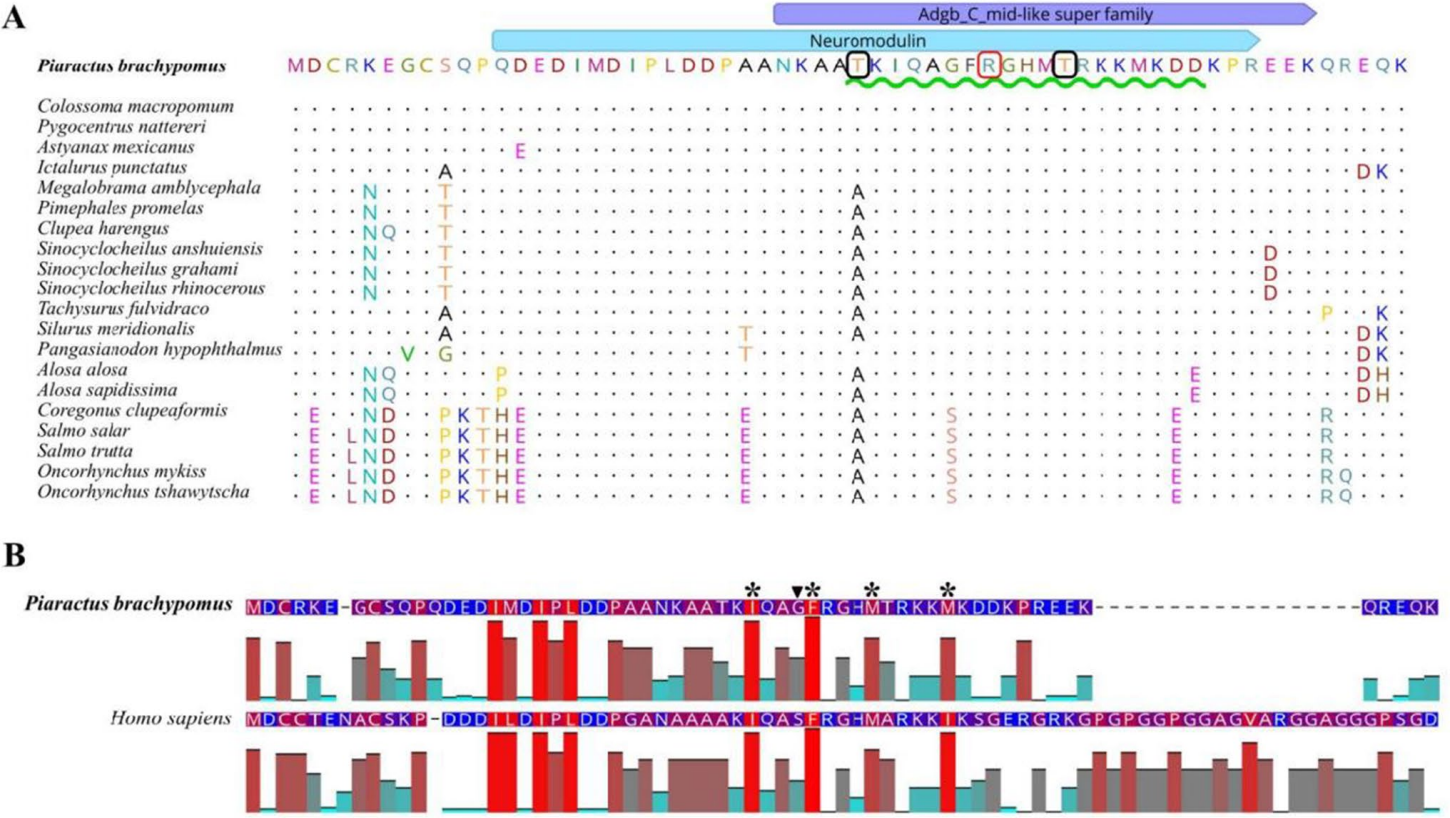
Comparative alignment of the amino acid sequence of *P. brachypomus* NRGN. **A** Multiple alignment of the amino acid sequences of NRGN from teleost fishes. *P. brachypomus* NRGN sequence was set as reference and conserved amino acids are indicated with dots, Adgb C mid-like (purple annotation) and neuromodulin (blue annotation). The IQ motif (I/L/V)QXXXRXXXX(R/K) essential consensus sequence in CaM binding is underlined in green. Essential Arg38 of the IQ motif highly conserved in teleost fish and mammals (red box). The predicted site for the PKC phosphorylation site is at position Thr31 and Thr42 (black box). **B** Pairwise alignment of NRGN from *P. brachypomus* and humans showing their hydrophobicity index. The serine located at position 36 is substituted by glycine in *P. brachypomus* (arrowhead). The key amino acids for binding with CaM are homologous in both sequences (asterisks), except for isoleucine located at position 46 substituted by methionine in *P. brachypomus *

*NRGN* full ORF from *P. brachypomus* contains 183 nucleotides that codes for 60 aa, with a molecular weight of 6945.87 Da (7 kDa), a theoretical pI of 7.77, total number of negatively charged (Asp + Glu) residues of 13 and positive (Arg + Lys) of 14. The index of instability was 43.34 and the amino acidic identity were from 68.33 to 100% among different fish species; the sequences with the highest identity with *P. brachypomus* were *Colossoma macropomum* and *Pygocentrus nattereri* with an identity of 100%. NRGN contains an IQ motif detected at the residues from Thr31 to Asp49 and a C-terminal middle region of androglobin (Adgb) super family (cl41701) from Asn27 to Lys55. Furthermore, two potential sites for PKC phosphorylation were identified (Fig. 1).

**Fig. 2:**
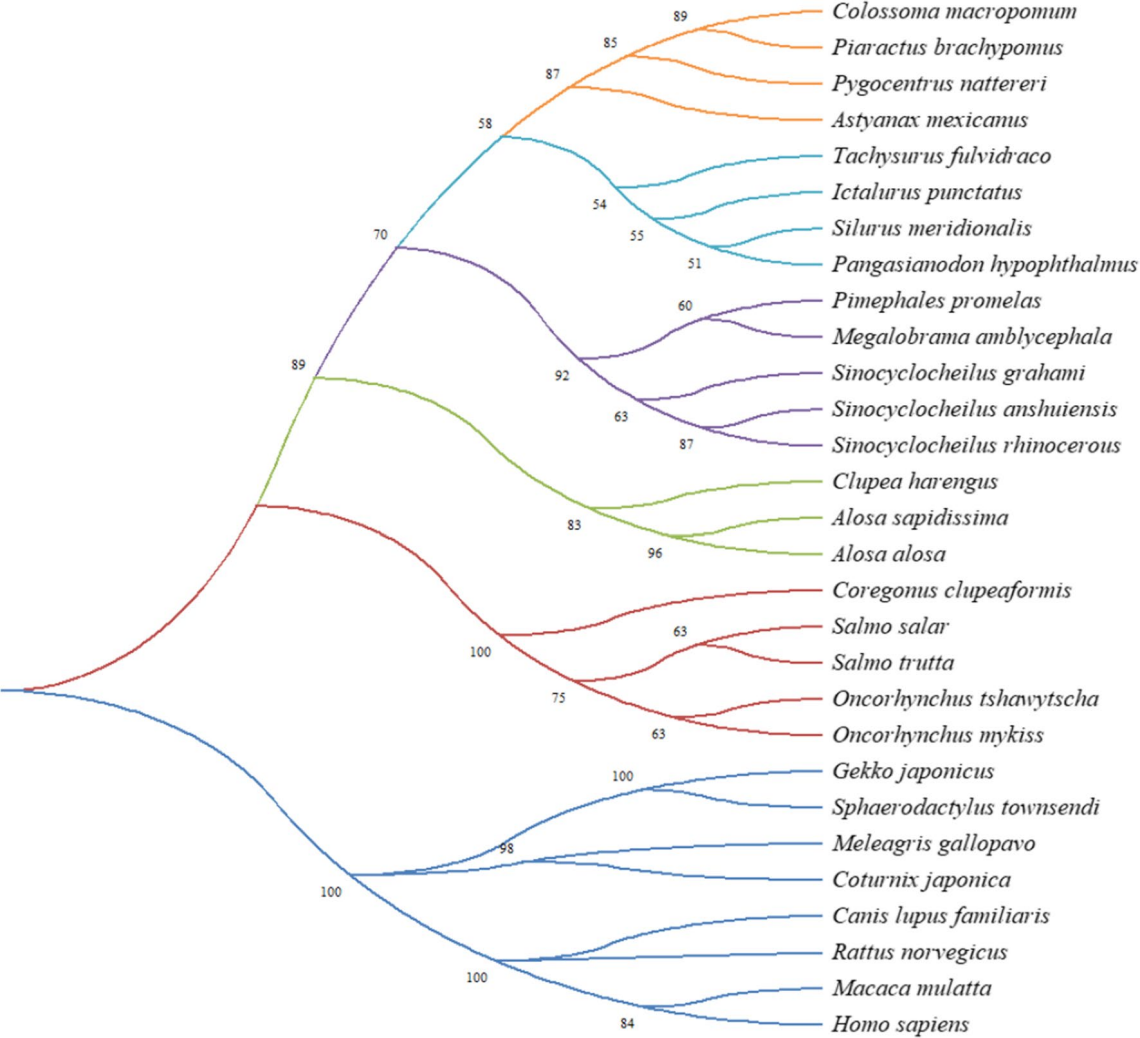
Phylogenetic tree (neighbor-joining) of amino acid sequences of NRGN from teleost fish, reptiles, birds, and mammals using the MEGA X. Orange clade: Characiformes; light blue clade: Siluriformes; purple clade: Cypriniformes; green clade: Clupeiformes; red clade: Salmoniformes; blue clade: reptiles, birds, and mammals. The values in the node branches indicate the percentage of replicate trees after bootstrap test (1000 replicates)

The phylogenetic analysis of the NRGN reveals six main clades. First clade corresponds to the order Characiformes (bootstrap = 87%), with an aa identity of 89% between *P. brachypomus* and *Colossoma macropomum*. Second clade corresponds to the order of the Siluriformes grouped in a branch with bootstrap of 53%, third clade belongs to Cypriniformes grouped with bootstrap of 70%, fourth clade was the Clupeiformes grouped with bootstrap of 89%, and the Salmoniformes grouped with bootstrap of 100% as fifth clade. Mammals-birds-reptiles constitute the sixth clade with bootstrap of 100% (Fig. 2).

The NRGN protein model showed differences in the amino acids of *P. brachypomus*; changes were found in six amino acids of the IQ motif compared to the human sequence. In the interaction of the CaM and NRGN complex, most of the key amino acids for the union of these could be observed; in the same way, the electrostatic surface was similar in *P. brachypomus* to humans, generating a pocket of negative charge, being essential for the binding of both.

### Expression of NRGN in Tissues of *P. brachypomus*

**Fig. 3:**
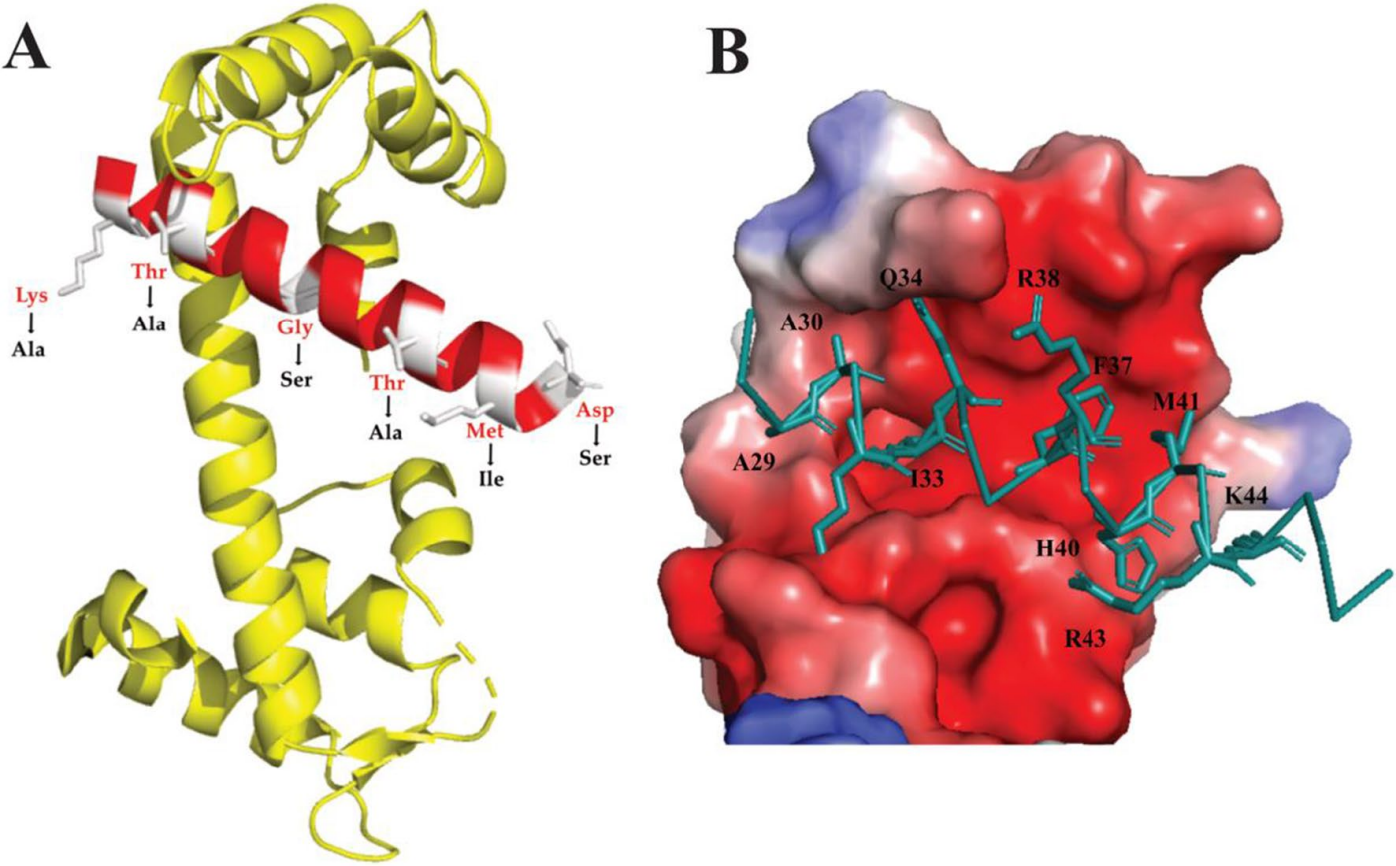
Modeling of the structure of the interaction of IQ of NRGN with the C-lobe of CaM. **A** NRGN-CaM complex, the amino acids in red belonging to *P. brachypomus* with the substitutions of the amino acids in black belonging to *Homo sapiens*. **B** The electrostatic surface potential of the C-lobe of CaM and IQ motif of NRGN. Key amino acids for the blinding of the complexes (negative charge, red; positive charge, blue)

**Fig. 4:**
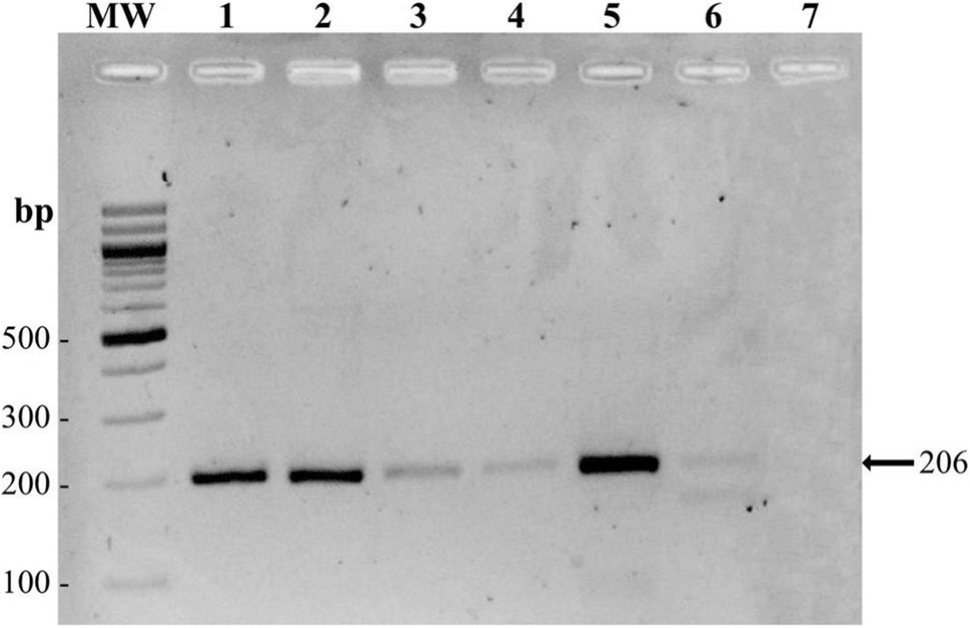
PCR amplification of *NRGN* ORF (206 bp) from cDNA samples of *Piaractus brachypomus*. MW: molecular weight marker 100 bp DNA ladder (New England Biolabs, USA), 1: brain, 2: gills, 3: liver, 4: blood, 5: head kidney, 6: spleen, 7: negative control (no template control). Agarose gel 2%

RT-PCR was performed using *NRGN*-specific primers to amplify the ORF with an amplicon of 206 bp. The ORF was amplified in cDNA samples from brain, spleen, liver, gills, blood, and head kidney (Fig. 3). Stronger bands were observed in the brain, gill, and head kidney (Fig. 4).

**Fig. 5:**
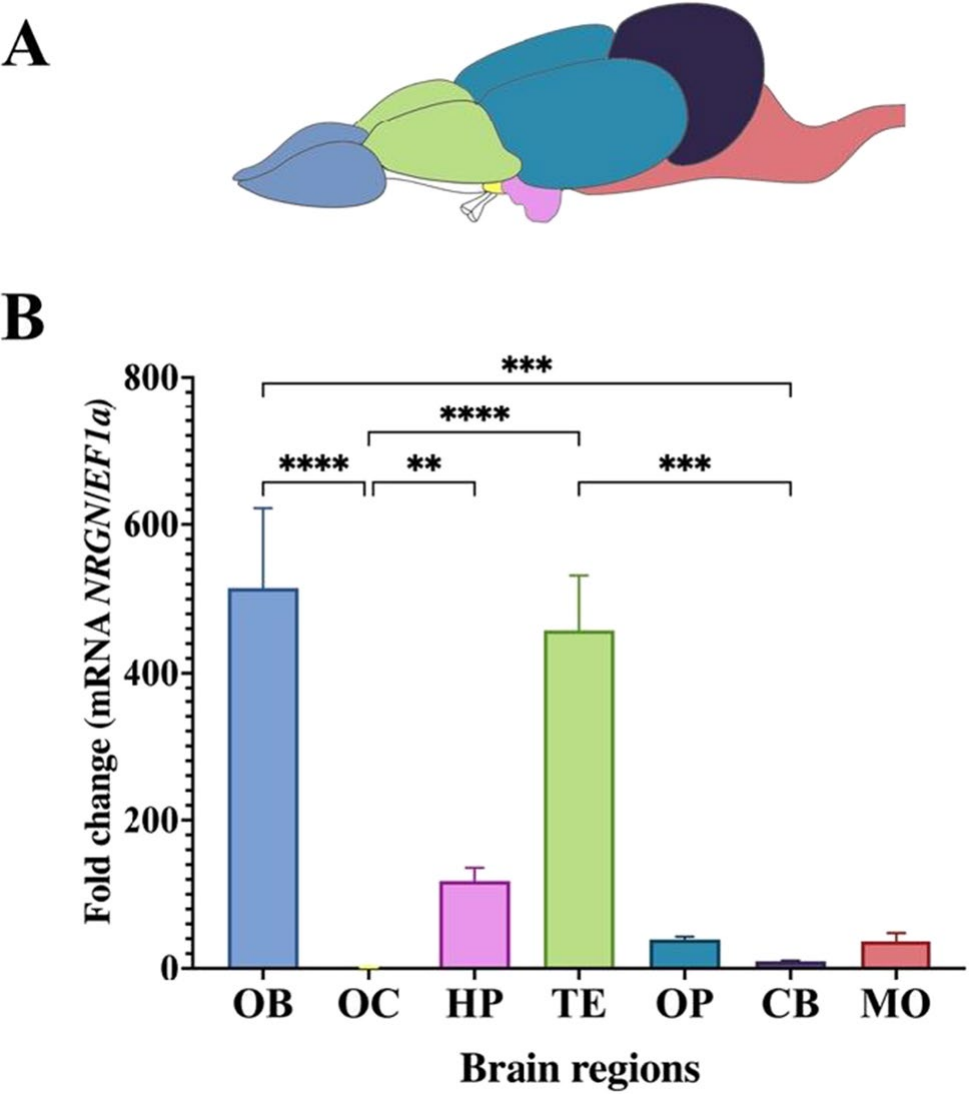
Expression of *NRGN* transcripts in brain regions by qPCR. **A** Color-coded illustration of *P. brachypomus* depicting regions of the brain. **B** Basal *NRGN* gene expression in brain tissues. OB olfactory bulb, OC optic chiasm, HP hypothalamus, TE telencephalon, OP optic bulb, CB cerebellum, MO medulla oblongata. **p < 0.01, ***p < 0.001, ****p < 0.0001

In brain regions, *NRGN* mRNA expression was present in all samples. The highest expression was found in the olfactory bulb and telencephalon, a lower expression was obtained in the hippocampus, optic bulb, and medulla oblongata, and the regions with the lowest *NRGN* expression were cerebellum and optic chiasm (Fig. 5).

### Expression Levels of *NRGN* in Organophosphate Sublethal Toxicity and Brain Injury Assays

**Fig. 6:**
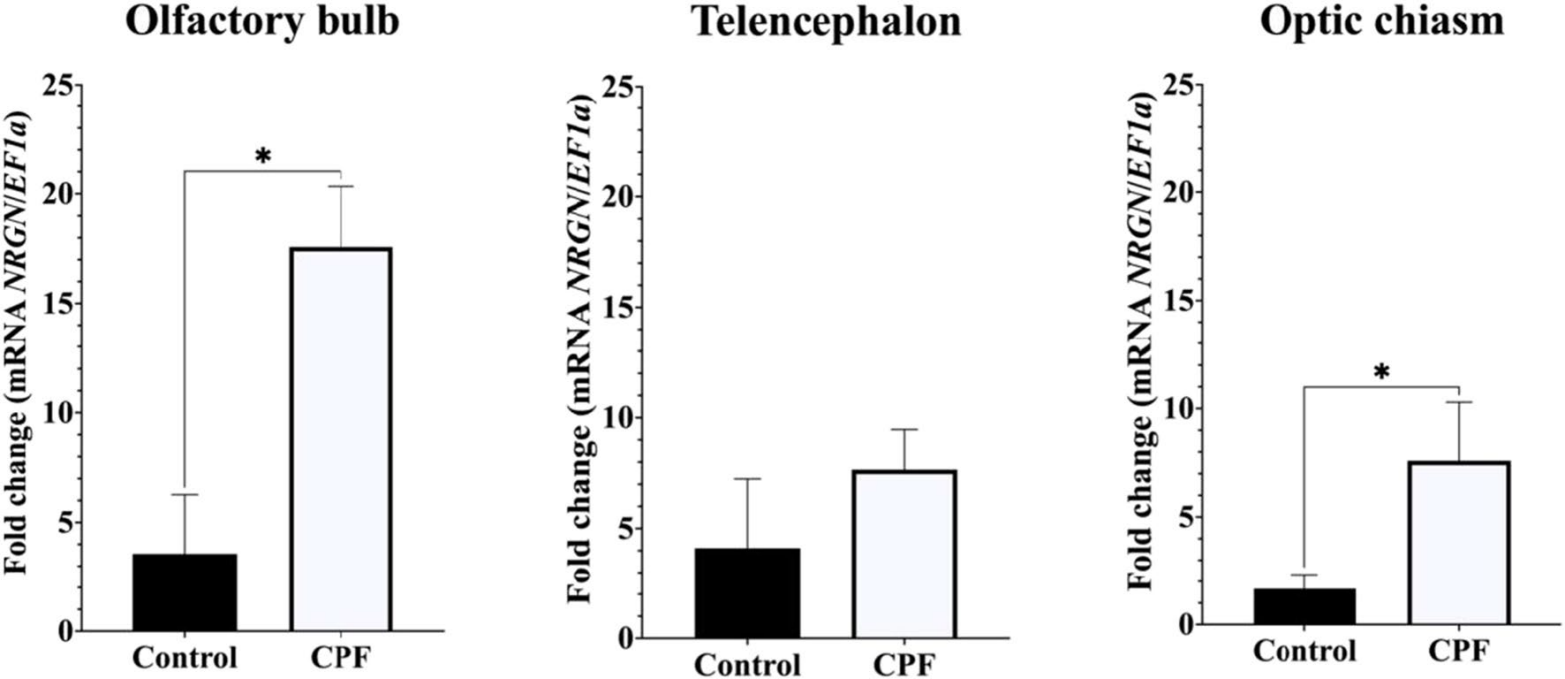
Relative gene expression of *NRGN* transcripts in brain regions (olfactory bulb, telencephalon, and optic chiasm) of red-bellied pacu exposed to sublethal concentrations of CPF. *p < 0.05

**Fig. 7:**
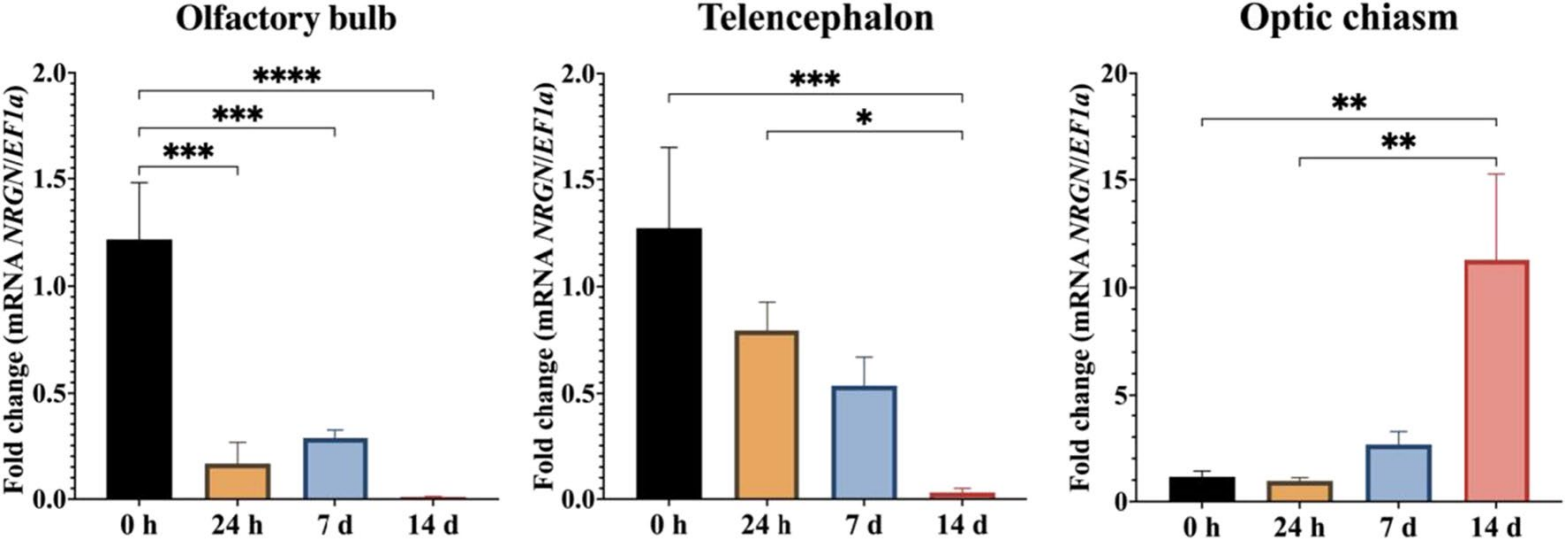
Relative gene expression of *NRGN* transcripts in brain regions (olfactory bulb, telencephalon, and optic chiasm) of red-bellied pacu after traumatic brain injury at 0 h, 24 h, 7 days, and 14 days. *p < 0.05, **p < 0.01, ***p < 0.001, ****p < 0.0001

qPCR analysis showed that *NRGN* was expressed in regions of the brain in both experimental models. The expression of *NRGN* transcripts in olfactory bulb (OB) and optic chiasm (OC) was significantly higher than telencephalon (TE) in fish exposed to CPF (*p* < 0.05) (Fig. 6). In brain injury assay, there were significant differences in all tissues. In OB, *NRGN* expression was downregulated at 24 h, 7 days (*p* < 0.001), and 14 days (*p* < 0.0001) compared with 0 h. Likewise, *NRGN* expression in TE was significantly higher at 0 h than 14 days (*p* < 0.001) and 24 h was significantly higher than 14 days (*p* < 0.05). Finally, OC showed a significantly higher expression of *NRGN* on 14 days than 0 h and 7 days (*p* < 0.01) (Fig. 7).

## Discussion

NRGN is a protein that has been reported in mammals and fish. In the present study, the cDNA coding for NRGN was described for the first time in *Piaractus brachypomus*, which was identified with 183 nucleotides that code for 60 aa, like other species of Characiformes such as *Astyanax mexicanus* (XP_015460274.1), *Pygocentrus nattereri* (XP_017552910.1), and *Colossoma macropomum* (XP_036435715.1). A molecular weight of 6945.87 Da (~ 7 kDa) was obtained, similar to the molecular weight reported in the rat of 7.6 kDa [26, 27]. The size of the protein may be essential to easily cross the blood–brain barrier and serve as a biomarker of brain damage [7].

The isoelectric point of NRGN was 7.77; biochemical studies found that the unions between NRGN and the multilamellar vesicles are generated in membranes with lipid acids such as phosphatidylserine, phosphatidylglycerol, phosphatidylinositol, or phosphatidic acid, which suggests the importance between the basic amino acids of NRGN and their interaction with lipids [28]. The calculated positive residues (Arg/Lys) were high in NRGN of *P. brachypomus*; this is related to the conserved regions of Arg that are surrounded by CaM negative residues, resulting in the binding of the IQ motif with CaM [20].

An IQ motif was detected between residues Thr31 to Asp49; IQ motifs are defined by a consensus sequence (I/L/V)QXXXRXXXX(R/K), which is essential for CaM binding [29]. In the case of *P. brachypomus*, key amino acids for NRGN/CaM binding were detected, such as Ile33, Phe37, Arg38, His40, Met41, Arg43, Lys44, Lys45, and Lys47 [20]. The interaction of Ser36 as a protein kinase C (PKC) phosphorylation site has been described in the IQ motif [30]; in our study, two phosphorylation sites for PKC were predicted by bioinformatics analyses of the protein; however, future analyses are required to confirm whether these predicted sites are functional and play a role in PKC phosphorylation. In the multiple sequence alignment, we identified a serine (Ser36) to glycine (Gly) substitution within the Characiformes group, including *P. brachypomus*. This substitution has previously been documented in *Danio rerio* and other teleost fishes [9]. When aligning the sequence of *P. brachypomus* with that of *Homo sapiens* (refer to Fig. 1B), similar levels of serine/glycine hydrophobicity were observed. However, studies conducted in *Xenopus* have reported that the phosphorylation of NRGN by PKC enhances the mobilization of intracellular Ca^2+^ and reduces the function of NRGN as a CaM buffer, therefore, mutations in the phosphorylation site with other amino acids such as Ala and Gly have been shown to prevent the phosphorylation of NRGN by PKC [31]. For this reason, further studies are needed to ascertain whether the substitution at the phosphorylation site in teleost fish affects NRGN phosphorylation in a similar manner.

The GRAVY index in NRGN was − 1.59, indicating that it is a hydrophobic protein. According to Kumar et al., NRGN has hydrophobic amino acids such as Ile33, Phe37, and Met41 which are involved with its binding ability [20]; in *P. brachypomus*, the homology was found in the amino acids reported.

### Phylogenetic Analysis

In the phylogenetic analysis of NRGN, a separation into six clades is evidenced, with a separation of mammals, birds, reptiles, and fish. *P. brachypomus* was placed within the teleost fish, specifically in the clade of the Characiformes. The difference between clades can be due to the evolutionary difference of the brains between them. In teleost fish, they have fluid-filled enclosures on the brain surface, in reptiles and birds they have a large and complex telencephalic structure with a dorsal ventricular crest absent in mammals, and mammals have developed a laminar architecture with neurons [32].

In NRGN, Clayton et al. found divergences in the C-terminus of the protein in canaries and mammals, which may deduce a development of different properties between them; in addition, it was found that NRGN is present in Purkinje cells in songbirds [33], a difference from the rodents and primates where it has been identified in embryonic stages, reflecting differences in physiology between mammals and birds [34]. In fish, NRGN was reported in all the main regions of the brain, with a wider distribution than that reported in mammals, finding NRGN in the interpeduncular nucleus, which is absent in mammals; these differences suggest a divergent evolution among these vertebrates [9].

### Expression of *NRGN* in Regions of Brain.

In the study, *NRGN* mRNA levels were found in all brain regions; however, there was higher expression in the OB and TE. High expressions of NRGN have been found in telencephalic areas, with specific postsynaptic localization in neurons of the OB, cerebral cortex, hippocampus, and amygdala [3, 35]. In teleost fish, the olfactory organ is composed of olfactory sensory neurons (OSNs) that extend their axons to the olfactory bulbs, in which glomeruli form, synapsing with bulbar mitral cells; these cells transmit their signals to the telencephalon processing areas, which is essential for odor-mediated behavior in fish [36]. Energetic GABA GC subsets located in the granule cell layer (GCL) have been found to express NRGN in murine models [37]. Additionally, the presence of NRGN has been reported in the axonal terminals of the glomeruli in *Danio rerio* [9]. The increased levels of *NRGN* mRNA in *P. brachypomus* can be related to its participation in synaptic plasticity and functionality of OB circuits.

The telencephalic areas in fish receive sensory signals and sources of spatial information which is useful for orientation and navigation [38]. In mammals, the hippocampus plays the role of navigation, memory, and signal recognition [39]. The telencephalic pallium of teleost fish is considered to contain regions homologous to the mammalian hippocampus, olfactory cortex, amygdala, and dorsal cortex [40]. As mentioned above, these same areas have the highest expression of NRGN; in addition, recent findings explain that NRGN may be involved in learning and memory and is highly expressed in telencephalic areas during these processes [41]. When measuring the basal levels of NRGN mRNA, it was found that the TE was significantly elevated in *P. brachypomus*; this may be due to the physiological behavior of the fish for orientation, danger signaling, spatial memory, etc.

### Expression of *NRGN* in CPF Sublethal Toxicity

CPF belongs to the group of organophosphate pesticides, which is one of the most used to control a wide range of insects [42]. CPF generates toxic effects by inhibiting acetylcholinesterase in nerve cells and neurotoxicity, neurobehavioral dysfunction, endocrine disruption, immunotoxicity, and oxidative stress have been reported in fish, specifically in *P. brachypomus* where brain lesions have been reported [22, 43]. Similarly, in humans, CPF causes neurotoxicity in prenatal development resulting in neurobehavioral deficits and slow brain growth [44].

As previously mentioned, the olfactory system is involved in fish behavior (e.g., feeding, orientation, defense), as well as in synaptic activities through dendritic spines. For this reason, the olfactory system is particularly vulnerable to exposure to pollutants, toxins, and pathogens, which makes olfactory plasticity crucial [36]. In organophosphates, CPF exposure affects cellular pathways, morphogenic genes, growth, and olfactory system processes in fish [45]. As a result, the olfactory system is equipped with a wide variety of mechanisms for neuroplasticity, remodeling, regeneration, and the generation of olfactory sensory neurons (OSNs) after damage [46, 47]. This mechanism has been reported in zebrafish, after acute exposure to heavy metals, a recovery is generated in the population of OSNs [48].

In the present study, an increase in the levels of *NRGN* mRNA in the OB was shown compared to the control group. This may be since the epithelium in fish presents continuous neurogenesis after injury and the overexpression of NRGN improves synaptic strength and reduces the threshold for LTP induction [49].

### Expression of *NRGN* in Brain Injury

TBI is one of the leading causes of death and disability in the world [50]. Similarly, TBI leads to neurodegenerative disorders, increasing the risk of generating neurological and behavioral deterioration [51]. For this reason, biomarkers in body fluids, extracellular vesicles, and miRNA have been studied to understand pathological mechanisms and perform early detection of TBIs [52]. In the present study, the levels of *NRGN* mRNA were evaluated in a TBI model at different times, in which a decrease in *NRGN* levels was found in TE and OB compared to the control group (0 h). These results are similar to those found by Svirsky et al., where at 24 h in the hippocampus there were significant reductions in NRGN protein levels, possibly due to a reduction in dendritic branches and low densities of dendritic spines after injury [8, 53]. In fish, the telencephalic areas may be homologous to the mammalian hippocampus [54] explaining the possible reduction of *NRGN* in TE found in *P. brachypomus*. Additionally, TBI has been associated with physical and sensory disturbances of the olfactory systems, including OB [55]. As previously mentioned, NRGN could be associated with plasticity mechanisms in the OB; therefore, the decrease in NRGN protein levels could be associated with damage to the olfactory system.

The presence of NRGN has been reported in different areas associated with the OC, such as the retina (photoreceptors, ganglion cells, etc.) and the optic tectum in zebrafish [9]. When an optic nerve lesion occurs, there is repopulation of astrocytes at 7 days, an increase in retinal microglia, and regeneration mechanisms of retinal ganglion cells (RGCs) [56]. Fishes have a greater capacity for regeneration of the visual system compared to mammals, since after an injury, the growth and regeneration of RGCs from the optic nerve to the optic chiasm has been evidenced at least 14 days after the injury [57]. These results coincide with those obtained in the experiment, where an overexpression of *NRGN* is evident 14 days after the lesion, probably due to the process of axonal regeneration and the generation of the synapse process of the visual system in *P. brachypomus*.

## Conclusion

NRGN from *P. brachypomus* showed a high identity with mammals and fish; in addition to this, a substitution of Ser36 for Gly36 was found in the phosphorylation site of PKC in teleost fish with levels of hydrophobicity similar to those of humans; however, further biochemical studies are required to determine if this substitution is part of PKC phosphorylation or plays a different role in molecular interactions with CaM. The high expression of the *NRGN* gene in some specific brain regions (e.g., OB) suggests an important role of NRGN in the pathophysiology of this tissue. This is the first study on the molecular characterization of neurogranin (*NRGN*) in *P. brachypomus* and its use as a biomodel in the study of brain lesions. Further studies are needed to understand the role of NRGN in synaptic plasticity in organophosphate poisoning and brain trauma, as well as the explanation of the structural and functional changes of NRGN binding with CaM in teleost fish and mammals.

## Data Availability

The dataset for this study is available from the corresponding author if requested.

## References

[1] Wang KK, Yang Z, Zhu T, Shi Y, Rubenstein R, Tyndall JA, Manley GT (2018). An update on diagnostic and prognostic biomarkers for traumatic brain injury. Expert Rev Mol Diagn.

[2] Çevik S, Özgenç M, Güneyk A, Evran Ş, Akkaya E, Çalış F, Kaynar M (2019). NRGN, S100B and GFAP levels are significantly increased in patients with structural lesions resulting from mild traumatic brain injuries. Clin Neurol Neurosurg.

[3] Represa A, Deloulme JC, Sensenbrenner M, Ben-Ari Y, Baudier J (1990). Neurogranin: immunocytochemical localization of a brain-specific protein kinase C substrate. J Neurosci.

[4] Petersen A, Gerges NZ (2015). Neurogranin regulates CaM dynamics at dendritic spines. Sci Rep.

[5] Agnello L, Gambino CM, Lo Sasso B, Bivona G, Milano S, Ciaccio AM, Piccoli T, La Bella V, Ciaccio M (2021). Neurogranin as a novel biomarker in Alzheimer’s disease. Lab Med.

[6] Koob AO, Shaked GM, Bender A, Bisquertt A, Rockenstein E, Masliah E (2014). Neurogranin binds α-synuclein in the human superior temporal cortex and interaction is decreased in Parkinson’s disease. Brain Res.

[7] Yang J, Korley FK, Dai M, Everett AD (2015). Serum neurogranin measurement as a biomarker of acute traumatic brain injury. Clin Biochem.

[8] Svirsky S, Henchir J, Li Y, Ma X, Carlson S, Dixon CE (2020). Neurogranin protein expression is reduced after controlled cortical impact in rats. J Neurotrauma.

[9] Alba-González A, Folgueira M, Castro A, Anadón R, Yáñez J (2022). Distribution of neurogranin-like immunoreactivity in the brain and sensory organs of the adult zebrafish. J Comp Neurol Jul.

[10] Zapata-Guerra NA, Rueda-Gómez DS, Lozano-Villegas KJ, Herrera-Sanchez MP, Uribe-García HF, Rondón-Barragán IS (2020). Menthol as anaesthetic for red-bellied pacu (*Piaractus brachypomus*) and its effect on HIF1a and GlucoR gene expression. Aqua Res.

[11] Holguín-Céspedes G, Céspedes-Rubio Á, Rondón-Barragán I (2022). First study on response of astrocytes in alevines of red-bellied pacu (*Piaractus*
*brachypomus*) to subchronic exposure to chlorpyrifos and trichlorfon. Vet World.

[12] Cruz-Méndez JS, Herrera-Sánchez MP, Céspedes-Rubio ÁE, Rondón-Barragán IS (2022). Molecular characterization of myelin basic protein a (mbpa) gene from red-bellied pacu (*Piaractus brachypomus*). J Genet Eng Biotechnol.

[13] Petano-Duque JM, Lozano-Villegas KJ, Céspedes-Rubio ÁE, Rondón-Barragán IS (2022). Molecular characterization of HEPCIDIN-1 (HAMP1) gene in red-bellied pacu (*Piaractus brachypomus*). Dev Comp Immunol.

[14] Naranjo Gómez JS, Vargas Rojas LF, Rondón Barragán IS (2013). Toxicidad aguda de cloruro de mercurio (HGCL2) en Cachama blanca: *Piaractus brachypomus* (Cuvier, 1818). Actualidades Biológicas.

[15] Geneious Prime (2023) version 2023.0.4. Biomatters Development Team. Available from: https://www.geneious.com. Accessed 15 Jan 2023.

[16] Blum M, Chang HY, Chuguransky S, Grego T, Kandasaamy S, Mitchell A, Nuka G, Paysan-Lafosse T, Qureshi M, Raj S, Richardson L, Salazar G, Williams L, Bork P, Bridge A, Gough J, Haft D, Letunic I, Marchler-Bauer A, Mi H, Finn R (2021). The InterPro protein families and domains database: 20 years on. Nucleic Acids Res.

[17] Lu S, Wang J, Chitsaz F, Derbyshire M, Geer R, Gonzales N, Gwadz M, Hurwitz D, Marchler G, Song J, Thanki N, Yamashita R, Yang M, Zhang D, Zheng C, Lanczycki C, Marchler-Bauer A (2020). CDD/SPARCLE: the conserved domain database in 202. Nucleic Acids Res.

[18] Blom N, Gammeltoft S, Brunak S (1999). Sequence and structure-based prediction of eukaryotic protein phosphorylation sites. J Mol Biol.

[19] DeLano W, Lam J (2005). PyMOL: a communications tool for computational models. Abstr Pap Am Chem Soc.

[20] Kumar V, Chichili VP, Zhong L, Tang X, Velazquez-Campoy A, Sheu FS, Seetharaman J, Gerges NZ, Sivaraman J (2018). Structural basis for the interaction of unstructured neuron specific substrates neuromodulin and neurogranin with calmodulin. Sci Rep.

[21] Kumar S, Stecher G, Li M, Knyaz C, Tamura K (2018). MEGA X: molecular evolutionary genetics analysis across computing platforms. Mol Biol Evol.

[22] Holguín-Céspedes G, Millan-Ocampo L, Mahecha-Méndez E, Cespedes Rubio A, Rondon Barragan I (2019). Toxicity assessment of chlorpyrifos in red-bellied pacu fingerlings (*Piaractus brachypomus*). RICA.

[23] Kishimoto N, Shimizu K, Sawamoto K (2012). Neuronal regeneration in a zebrafish model of adult brain injury. Dis Model Mech.

[24] CCAC (2010). CCAC guidelines on: euthanasia of animals used in science.

[25] Livak K, Schmittgen T (2001). Analysis of relative gene expression data using real-time quantitative PCR and the 2(-delta delta C(T)) method. Methods (San Diego, Calif.).

[26] Huang K, Huang F, Chen H (1993). Characterization of a 7.5-kDa protein kinase C substrate (RC3 protein, neurogranin) from rat brain. Arch Biochem Biophys.

[27] Watson J, Battenberg E, Wong K, Bloom F, Sutcliffe J (1990). Subtractive cDNA cloning of RC3, a rodent cortex-enriched mRNA encoding a novel 78 residue protein. J Neurosci Res.

[28] Gerendasy D, Sutcliffe J (1997). RC3/neurogranin, a postsynaptic calpacitin for setting the response threshold to calcium influxes. Mol Neurobiol.

[29] Bähler M, Rhoads A (2002). Calmodulin signaling via the IQ motif. FEBS Lett.

[30] Prichard L, Deloulme J, Storm D (1999). Interactions between neurogranin and calmodulin in vivo. J Biol Chem.

[31] Cohen RW, Margulies JE, Coulter PM, Watson JB (1993). Functional consequences of expression of the neuron-specific, protein kinase C substrate RC3 (neurogranin) in Xenopus oocytes. Brain Res.

[32] Sarnat H, Netsky M (2002). When does a ganglion become a brain? Evolutionary origin of the central nervous system. Semin Pediatr Neurol.

[33] Clayton D, George J, Mello C, Siepka S (2009). Conservation and expression of IQ-domain-containing calpacitin gene products (neuromodulin/GAP-43, neurogranin/RC3) in the adult and developing oscine song control system. Dev Neurobiol.

[34] Singec I, Knoth R, Ditter M, Volk B, Frotscher M (2004). Neurogranin is expressed by principal cells but not interneurons in the rodent and monkey neocortex and hippocampus. J Comp Neurol.

[35] Álvarez-Bolado G, Rodríguez-Sánchez P, Tejero-Díez P, Fairén A, Díez-Guerra FJ (1996). Neurogranin in the development of the rat telencephalon. Neuroscience.

[36] Calvo-Ochoa E, Byrd-Jacobs C (2019). The olfactory system of zebrafish as a model for the study of neurotoxicity and injury: implications for neuroplasticity and disease. Int J Mol Sci.

[37] Gribaudo S, Saraulli D, Nato G, Bonzano S, Gambarotta G, Luzzati F, Costanzi M, Peretto P, Bovetti S, De Marchis S (2021). Neurogranin regulates adult-born olfactory granule cell spine density and odor-reward associative memory in mice. Int J Mol Sci.

[38] Fotowat H, Lee C, Jun JJ, Maler L (2019) Neural activity in a hippocampus-like region of the teleost pallium is associated with active sensing and navigation. eLife 8:e44119. 10.7554/eLife.44119 10.7554/eLife.44119 PMC6469930 30942169

[39] Hartley T, Lever C, Burgess N, O'Keefe J (2013). Space in the brain: how the hippocampal formation supports spatial cognition. Philos Trans Royal Soc.

[40] Rodríguez F, Quintero B, Amores L, Madrid D, Salas-Peña C, Salas C (2021). Spatial cognition in teleost fish: strategies and mechanisms. Animals.

[41] Díez-Guerra FJ (2010). Neurogranin, a link between calcium/calmodulin and protein kinase C signaling in synaptic plasticity. IUBMB Life.

[42] Wołejko E, Łozowicka B, Jabłońska-Trypuć A, Pietruszyńska M, Wydro U (2022). Chlorpyrifos occurrence and toxicological risk assessment: a review. Int J Environ Res Public Health.

[43] Olsvik PA, Berntssen MHG, Søfteland L, Sanden M (2019). Transcriptional effects of dietary chlorpyrifos-methyl exposure in Atlantic salmon (*Salmo salar*) brain and liver. Comp Biochem Physiol. Part D, Genomics & proteomics.

[44] Rauh V, Arunajadai S, Horton M, Perera F, Hoepner L, Barr DB, Whyatt R (2011). Seven-year neurodevelopmental scores and prenatal exposure to chlorpyrifos, a common agricultural pesticide. Environ Health Perspec.

[45] Tilton FA, Tilton SC, Bammler TK, Beyer RP, Stapleton PL, Scholz NL, Gallagher EP (2011). Transcriptional impact of organophosphate and metal mixtures on olfaction: copper dominates the chlorpyrifos-induced response in adult zebrafish. Aquat Toxicol.

[46] Bayramli X, Kocagöz Y, Sakizli U, Fuss SH (2017). Patterned arrangements of olfactory receptor gene expression in zebrafish are established by radial movement of specified olfactory sensory neurons. Sci Rep.

[47] Kizil C, Kaslin J, Kroehne V, Brand M (2012). Adult neurogenesis and brain regeneration in zebrafish. Dev Neurobiol.

[48] Ma EY, Heffern K, Cheresh J, Gallagher EP (2018). Differential copper-induced death and regeneration of olfactory sensory neuron populations and neurobehavioral function in larval zebrafish. Neurotoxicology.

[49] Zhong L, Brown J, Kramer A, Kaleka K, Petersen A, Krueger JN, Florence M, Muelbl MJ, Battle M, Murphy GG, Olsen CM, Gerges NZ (2015). Increased prefrontal cortex neurogranin enhances plasticity and extinction learning. J Neurosci.

[50] Mollayeva T, Mollayeva S, Colantonio A (2018). Traumatic brain injury: sex, gender and intersecting vulnerabilities. Nat Rev Neurol.

[51] Brett BL, Gardner RC, Godbout J, Dams-O'Connor K, Keene CD (2022). Traumatic brain injury and risk of neurodegenerative disorder. Biol Psychiatry.

[52] Ghaith HS, Nawar AA, Gabra MD, Abdelrahman ME, Nafady MH, Bahbah EI, Ebada MA, Ashraf GM, Negida A, Barreto GE (2022). A literature review of traumatic brain injury biomarkers. Mol Neurobiol.

[53] Gao X, Deng P, Xu ZC, Chen J (2011). Moderate traumatic brain injury causes acute dendritic and synaptic degeneration in the hippocampal dentate gyrus. PLoS ONE.

[54] Saito K, Watanabe S (2006). Deficits in acquisition of spatial learning after dorsomedial telencephalon lesions in goldfish. Behav Brain Res.

[55] Marin C, Langdon C, Alobid I, Mullol J (2020). Olfactory dysfunction in traumatic brain injury: the role of neurogenesis. Curr Allergy Asthma Rep.

[56] Fague L, Liu YA, Marsh-Armstrong N (2021). The basic science of optic nerve regeneration. Ann Trans Med.

[57] Diekmann H, Kalbhen P, Fischer D (2015). Characterization of optic nerve regeneration using transgenic zebrafish. Front Cell Neurosci.

